# Parents’ Perspectives on the Use of Virtual Reality in Pediatric Chronic Pain Management: Qualitative Study

**DOI:** 10.2196/74082

**Published:** 2025-08-15

**Authors:** Lindsay Sullivan, Harrison Vriese, Ellie Ferguson, Megan Armstrong, Hannah Williams, Kathleen Lemanek, Sharon Wrona, Lauren Renner, Lindsey Vater, Henry Xiang

**Affiliations:** 1 Division of Health Sciences, School of Health and Rehabilitation Sciences, College of Medicine, The Ohio State University, 333 W 10th Ave, Columbus, OH, 43210, United States, 1 6148140393; 2 Center for Injury Research and Policy, The Abigail Wexner Research Institute, Nationwide Children's Hospital, Columbus, OH, United States; 3 Center for Pediatric Trauma Research, The Abigail Wexner Research Institute, Nationwide Children's Hospital, Columbus, OH, United States; 4 Department of Pediatrics, College of Medicine, The Ohio State University, Columbus, OH, United States; 5 Department of Pediatric Psychology and Neuropsychology, Nationwide Children's Hospital, Columbus, OH, United States; 6 Comprehensive Pain and Palliative Services, Nationwide Children's Hospital, Columbus, OH, United States

**Keywords:** adolescents, children, chronic pain, management, parents, virtual reality

## Abstract

**Background:**

Virtual reality (VR) technology holds significant potential for chronic pain management in children and adolescents by providing an alternative and complementary approach to traditional methods of alleviating pain and improving quality of life. Parents play an important role in the successful adoption of VR technologies for children, influencing how children accept, use, and benefit from it. However, little is known about parents’ views on integrating VR technology into pediatric and adolescent chronic pain management.

**Objective:**

This study aimed to better our understanding of parents’ perspectives regarding the integration of VR technology into pediatric and adolescent chronic pain management—including barriers, facilitators, and recommendations for future VR technologies.

**Methods:**

Semistructured interviews were conducted with parents of children with chronic pain between March and November 2024. Key aspects of the discussion centered on the acceptability, barriers, and enablers of integrating VR technology into pediatric and adolescent chronic pain management. Interviews were audio-recorded, transcribed, and analyzed through inductive thematic analysis.

**Results:**

We conducted 12 interviews. We identified four broad themes from the interview data: (1) views toward integrating VR technology into chronic pain management (perceived benefits, distraction, and redirection); (2) barriers to using VR technology for chronic pain management (accessibility, complexity, discomfort, and symptom exacerbation); (3) facilitators of integrating VR technology into chronic pain management (addressing financial barriers, integrating VR technology into clinical care, establishing evidence of effectiveness and showcasing positive patient experiences); and (4) recommendations for program content and features (relaxation and mindfulness, physical activity, customization, and social connection).

**Conclusions:**

Our findings underscore the perceived benefits of integrating VR technology into pediatric and adolescent chronic pain management to enhance physical, social, and mental health and well-being. However, there are several potential challenges that need to be addressed to improve the accessibility of VR technology for use in pediatric and adolescent chronic pain management. Our findings yielded several practical suggestions to guide the development of effective and equitable VR technology for chronic pain management in children and adolescents.

## Introduction

Chronic pain, defined as pain persisting for 3 months or longer [1], is both prevalent and burdensome in children and adolescents [1]. Evidence suggests that approximately 1 in 5 children and adolescents experience chronic pain [1,2]. Chronic pain in children and adolescents may be the consequence of ongoing or progressive disease (eg, sickle cell disease, cancer, and treatment-related pain), injury (eg, burns and complex regional pain syndrome), or an adjustment disorder (eg, somatoform disorder and pain disorder) [3]. Other children and adolescents experience nonspecific or unexplained chronic pain, such as headache, recurrent abdominal pain, or fibromyalgia [3]. Chronic pain can adversely affect an individual’s physical health by leading to functional limitations such as difficulties in performing activities of daily living, sleep disturbances, and impaired participation in sports and physical activity [2]. In addition to physical discomfort and limitations, chronic pain can have negative and long-lasting implications on one’s mental health and quality of life [1,2]. Chronic pain can also interfere with school attendance and inhibit engagement in social activities in children, negatively impacting their social health and wellness [1,2].

Managing pediatric and adolescent chronic pain is inherently challenging due to difficulties in pain communication, variability in developmental and cognitive factors, and the multifactorial nature of pain itself [4-6]. Traditional management approaches for chronic pain in children and adolescents include pharmacotherapy, physical therapy, and psychological interventions [6,7]. However, these approaches often require substantial resources, including time and accessibility to multidisciplinary teams [4,6,7]. These challenges underscore the necessity for more holistic and integrated treatment strategies tailored to the unique needs of children and adolescents with chronic pain. Moreover, despite recent evidence demonstrating the importance of a biopsychosocial model in understanding and managing pediatric chronic pain [8], many health care professionals report limited training and knowledge in assessing and managing chronic pain in children, contributing to persistent gaps in care [2]. Incorporating more interdisciplinary, evidence-based rehabilitation programs into treatment plans for pediatric and adolescent populations with chronic pain has been advocated to improve both physical and psychological outcomes, including pain management, functional ability, and overall quality of life [4,6].

Digital health interventions have emerged as promising complements or alternatives to traditional treatments for chronic conditions in children and adolescents, offering flexible, scalable, and child-centered support that can be integrated into daily life [9,10]. Digital platforms, ranging from mobile apps and web-based programs to telehealth consultations and wearable devices, may increase accessibility, reduce health care burdens, enhance treatment adherence, and potentially reduce therapy costs [9,11]. While evidence suggests that digital health interventions yield small-to-moderate benefits in improving self-efficacy and some disease-related outcomes for youth with chronic conditions, evidence on the impact of these interventions on other mental health and quality-of-life outcomes is limited [9].

Within this digital health landscape, virtual reality (VR) shows promise for pain management in children. Prior studies demonstrate medium to large effect sizes of VR technologies in reducing pain and anxiety during various medical procedures in children, particularly burn care and venous access procedures [12]. Additional benefits of VR technology for pain management include improved coping skills, enhanced functioning, and rehabilitative support [10,13,14]. Most existing research on VR technologies focuses on acute pain management in children, with limited studies examining the benefits of use in children and adolescents with chronic pain [10,13,14]. Early pilot data indicate that VR technologies are feasible and safe and may facilitate meaningful functional gains in chronic pain rehabilitation settings for youth [10].

Despite growing enthusiasm for the use of VR in pediatric and adolescent pain management, evidence of its effectiveness in chronic pain management is limited. A 2025 systematic review mapped just 6 chronic pain trials out of 90 pediatric VR studies for pain management (~7%) [15]. Most applications of VR in pediatric chronic pain management have focused on physical therapy, with the goal of improving physical function [10,16,17]. Many broader opportunities for VR to restore physical, psychological, and social functioning and create engaging, empowering, and therapeutic experiences for children with chronic pain remain underdeveloped. Further, heterogeneity in hardware, software, and session parameters and more than 30 distinct outcome measures make it difficult to compare the effectiveness of VR technology across studies [15]. Methodological quality is modest at best, as 74% of studies carried moderate bias, while 19% of studies had a high risk of bias [15]. Safety data is similarly patchy; although no serious events were identified, minor cybersickness symptoms such as nausea or dizziness occurred in up to 17% of pediatric users, and adverse event reporting was generally poor, leaving longer-term risks unclear [15,18]. Collectively, these gaps call for larger, multisite randomized controlled trials with standardized protocols, extended follow-up, and core outcome sets that capture functional and psychosocial domains alongside pain.

Parents’ and family caregivers’ perceptions of adopting VR technologies for pediatric and adolescent chronic pain management are crucial for successful implementation yet remain largely unexplored. Understanding their perspectives is essential to designing effective, user-centered digital interventions that align with their needs and concerns. This paper aims to better our understanding of parents’ perspectives regarding the integration of VR technology into pediatric and adolescent chronic pain management—including barriers, facilitators, and recommendations for future VR technologies. We specifically focused on parents because they often serve as key decision-makers in pediatric health care. Our findings will inform the refinement and development of VR technologies for chronic pain management and support efforts to make pediatric and adolescent pain management more accessible, personalized, and effective.

This study was guided by the technology acceptance model (TAM), which aims to understand predictors of individuals’ acceptance or rejection of emerging technologies. The TAM, developed by Davis in 1989, is an extension of the theory of reasoned action [19]. The TAM has gained considerable prominence in explaining the acceptance of technologies in the health care context and has been validated by extensive empirical support [20,21]. According to TAM, attitudes towards the acceptance or rejection of technology, subsequent behavioral intentions, and actual usage of novel technology are primarily determined by two factors: (1) perceived usefulness (one’s belief about whether the technology will improve their performance) and (2) perceived ease of use (one’s belief about how effortless using the technology will be) [19]. The TAM posits that the stronger the intention to use technology, the more likely an individual will be to use the technology. By applying the TAM framework, this study will generate meaningful insights to inform the refinement, development, and adoption of VR technologies for chronic pain management and support efforts to make pediatric and adolescent pain management more accessible, personalized, and effective.

## Methods

### Overview

Qualitative methods are particularly valuable in this context as they provide rich, in-depth insights into personal experiences and beliefs that quantitative approaches may not fully capture [22]. This study used a phenomenological approach to identify and examine perceived barriers and facilitators influencing the adoption of VR for chronic pain management [23]. The study was conducted and reported in accordance with the RATS (Relevance, Appropriateness, Transparency, Soundness) guidelines for qualitative research [24].

### Research Design

We interviewed the parents of children and adolescents aged 10-17 years with chronic pain. Chronic pain was defined as pain for a period of at least 3 months. Parents were eligible for the study if they were English-speaking and their child was receiving treatment for chronic pain at the time of enrollment. We used a variety of methods to recruit parents through the Comprehensive Pain Services Department at a large Midwest Children’s Hospital. Eligible parents were given an information sheet by their health care team at a clinical visit containing important details about the study—including the study aims, methods, and time commitment. Parents who were interested in participating were directed to the web-based consent form and demographics survey. Parents who completed the web-based consent form and demographics survey were then contacted by email by a member of the research team to schedule the interview. Researchers also recruited parents directly in clinic waiting areas. Interested parents recruited in clinic waiting rooms completed the web-based consent form and demographics survey and then participated in the interview in a private room. Although the sample was recruited from a variety of sources, all the parents had a child currently receiving comprehensive care for chronic pain. We interviewed 1 parent per family, except for 1 case where both parents wanted to participate; the family designated which parent would participate. We completed 12 interviews between March and November 2024.

### Data Collection and Interviews

The qualitative data were collected through semi-structured interviews conducted by the first author. The interview guide covered topics in the following main domains: (1) history of chronic pain, (2) experiences with chronic pain, (3) management of chronic pain, and (4) VR technologies for chronic pain management. All questions posed were broad and open-ended (see Textbox 1). Although the interview guide covered multiple topics, we conducted the interviews with sensitivity to each participant’s narrative. This flexible approach allowed us to prompt and explore topics spontaneously, thereby enhancing the richness of our findings. Only the qualitative information on the views toward VR technologies for chronic pain management is presented in this paper (see Textbox 1). Participants’ understanding of VR was based solely on prior knowledge and experiences with VR technology. Participants were not shown examples of VR nor provided with descriptions of potential applications of VR to pediatric and adolescent chronic pain management. Additional qualitative information gathered in these interviews is subject to further analyses beyond the scope of this paper.

The interviews lasted between 30 and 90 minutes. Most interviews took place via phone, although a few took place in person in a quiet, private area. Parents were assured of the confidentiality of their responses. They were also informed that participation in the study was completely voluntary, and they could withdraw at any time. The protocol was reviewed and approved by the institutional review board at The Ohio State University. All parents provided web-based informed consent.

Textbox 1. **Questions for the individual interview.**

1. Do you think a VR program that helps your child manage their chronic pain at home would be useful? Why or why not?
2. Has your child ever used a VR program or application, such as a mobile app or web-based platform, to help them manage their pain?
3. If you were to create a VR program for children with chronic pain, what would it look like?
4. What are potential barriers, if any, to VR program use among children and adolescents with chronic pain? Would anything promote the use of a VR program among children and adolescents with chronic pain?

### Demographic Characteristics

Before the interview was completed, the participants were asked to complete a brief web-based survey asking for information about the demographic characteristics of the parents and their children—including the age and sex of both parent and child; how long the child has experienced chronic pain; types of pain experienced by the child; the child’s diagnosis, if any; and their access to internet and technology (eg, computer, smartphone, and VR) at home.

### Data Analysis

All interviews were audio recorded and transcribed verbatim. Transcribed data were deidentified with subject identifiers assigned to each parent. Data were analyzed using an inductive thematic analysis approach [25]. Inductive thematic analysis enables researchers to identify themes from the data rather than applying a predefined framework or theory. We conducted the inductive thematic analysis in three steps: (1) immersion in the topic, (2) identification of possible themes, and (3) reviewing of themes. Initial thematic analysis was conducted independently by 2 research team members (LS and HV). Each researcher (LS and HV) read and reread the transcripts to familiarize themselves with the data and note their initial impressions. The researchers independently coded the transcripts line by line, identifying initial descriptive codes and themes. The researchers then discussed the themes identified and developed a master codebook with themes and definitions so that coding was consistent across interviews. Each researcher then coded each interview using the master codebook. Throughout these processes, the research team met regularly to consult about codebook development, discuss discrepancies, and recode transcripts as needed until final coding was reached. Minor discrepancies were resolved by discussion and consensus between the 2 researchers and validated by another member of the research team (EF). Data saturation was achieved after 12 interviews, aligning with prior qualitative research findings indicating that saturation typically occurs within 10‐12 interviews in homogeneous samples [26,27]. The final coded data was reviewed and validated by another team member (EF). The findings reported here answer the following key research questions: (1) What are parents’ views toward the integration of VR into chronic pain management for children and adolescents? (2) What factors would hinder or promote the use of VR technologies for chronic pain management in children and adolescents?

### Ethical Considerations

This study was examined and deemed exempt from review by the Ethics Committee of The Ohio State University (2023E1333). All participants provided web-based informed consent prior to data collection. Participation was voluntary. The transcripts were deidentified for data analysis. Participants received a US $25 Amazon gift card for their participation in the study.

## Results

### Demographic Characteristics of the Study Participants

We interviewed 13 parents of children with chronic pain. Demographic characteristics of parents and their children are presented in Table 1. Most parents were female (n=10, 76.9%) as was their child with chronic pain (n=9, 75.0%). The mean age of the children with chronic pain was 15.0 (SD 2.49; range 10‐17) years. Most children (n=10, 83.3%) experienced multiple pain complaints. The most common type of pain complaints were headaches, including migraine (n=8, 66.7%), followed by joint (n=6, 50.0%), back (n=5, 41.7%), and leg pain (n=5, 41.7%). Of the 12 children with chronic pain, only 5 (41.7%) had a medical diagnosis for the underlying cause of their pain. Children had been experiencing their pain for a period of 6 months to 9 years. Only 1 participant had a history of using VR for chronic pain management.

**Table 1.** Demographic characteristics of participating parents and their child with chronic pain.

Demographic characteristics	Values
Parent	
Sex	
Female, n (%)	10 (76.9)
Male	3 (23.1)
Age, years, mean (SD)	44.9 (5.8)
Prior use of VR for pain management, n (%)	
Yes	1 (7.7)
No	12 (92.3)
Child	
Sex, n (%)	
Female	9 (75)
Male	3 (25)
Age, years, mean (SD)	15.0 (2.5)
Duration of chronic pain, years, mean (SD)	2.8 (2.2)
Type of chronic pain, n (%)	
Abdominal	4 (33.3)
Arm	2 (16.7)
Back	5 (41.7)
Body	4 (33.3)
Chest	3 (25)
Headaches, including migraine	8 (66.7)
Joint	6 (50)
Leg	5 (41.7)
Neck	3 (25)
Pelvic	3 (25)
Confirmed diagnosis for the underlying cause of their chronic pain, n (%)	
Yes	5 (41.7)
No	7 (58.3)

### Interview Data

**Figure 1.**
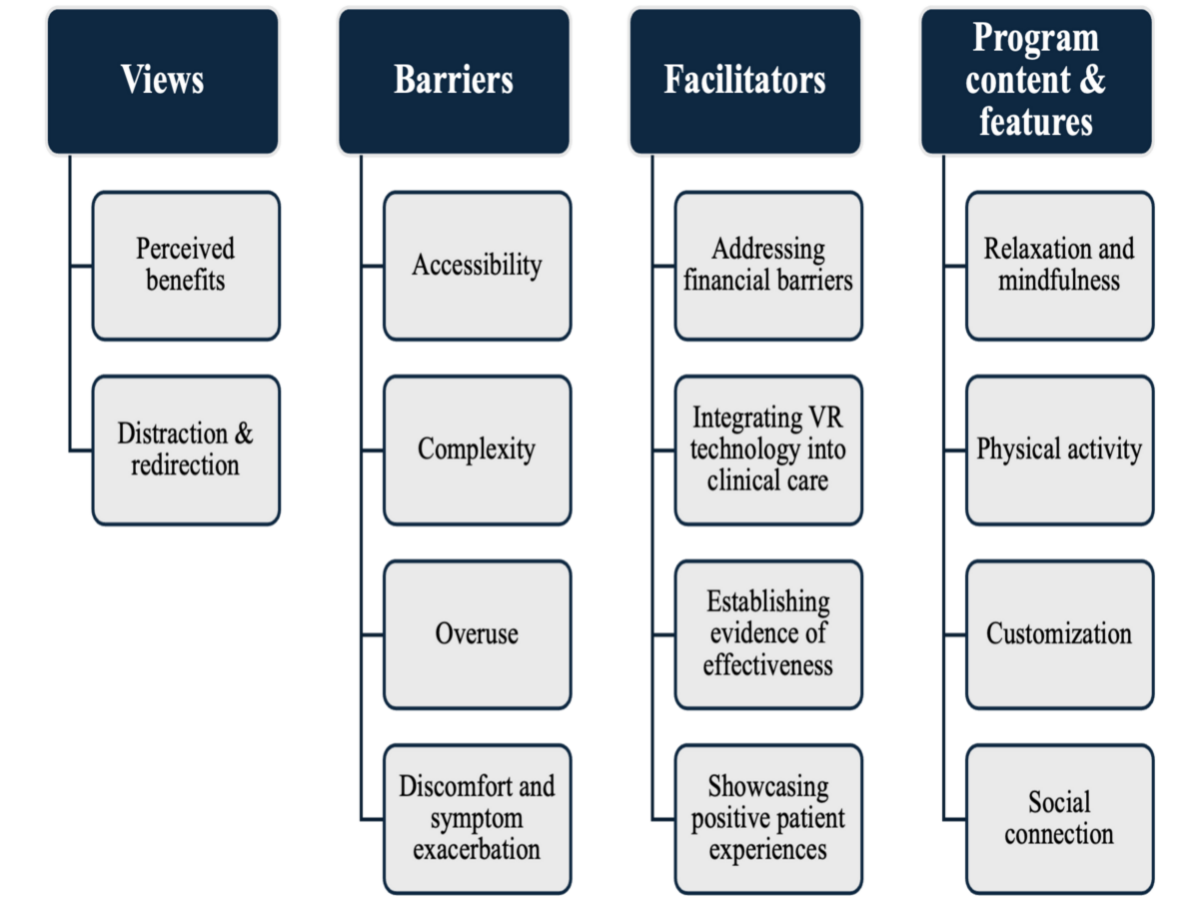
Themes and subthemes from parents’ views toward the integration of VR into chronic pain management for children and adolescents. VR: virtual reality.

We identified four broad themes from the interviews: (1) views toward integrating VR technology into chronic pain management, (2) barriers to using VR technology for chronic pain management, (3) facilitators of integrating VR technology into chronic pain management, and (4) recommendations for program content and features (Figure 1). In total, 2-4 subthemes for each theme were identified and are described in greater detail below, with illustrative quotes in text and additional quotes provided in Table 2.

**Table 2.** Parents’ perspectives regarding the integration of VR technology into pediatric chronic pain management.

Themes and subthemes	Quotes
Theme: Views	
Perceived effectiveness	“Yeah, I think anything that would help them with the mind-body connection, like reducing their level of anxiety about the pain would be awesome.” (Parent 1) "So, for one, because I think it would be very useful because as a parent, when your child is going through chronic pain and you do everything, you go through the checklist, and you have nothing else to check off.” (Parent 11)
Distraction and redirection	“Just anything with helping them… to help them feel better… not thinking about pain all the time.” (Parent 12) "I think that getting the escape away from the body would help… I think that kind of putting on a VR headset kind of makes you feel like you’re out of your body. Yeah. And so that would make you feel like you can’t feel that pain.” (Participant 7)
Theme: Barriers	
Accessibility	*“*Definitely cost. It is very expensive.” (Participant 2) “The only thing that would be financially. If they can afford the stuff and the internet.” (Parent 11)
Complexity	“…making sure that it’s something that’s easy to use that a child could set up without the help of their parent.” (Parent 1) “…if you’re not technology savvy, it can be a little confusing to figure it out… there would definitely need to be some… training for parents…” (Parent 2)
Overuse	“The only barrier I see is if parents have an issue, you know, screen time or whatever.” (Participant 9) “I think you’re gonna have to sell it to the parents more than your kids.” (Parent 11)
Discomfort and symptom exacerbation	“If it causes more pain with movement or certain moves they have to do to get through the game. I think it would probably just depend on where the pain is and what type of game it is.” (Parent 4) “She says she can’t because the flashy lights give her migraine and okay, you know, to make her dizzy.” (Parent 10)
Theme: Facilitators	
Addressing financial barriers	“Or if it’s something that insurance could even, I mean cover at some point like oh here’s this basic equipment.” (Parent 6) “If you could rent them or…, do you have to have your own system?” (Parent 12)
Integrating VR technology into clinical care	"I feel like if doctor’s offices or if it was part of pain management clinics, or again, if it was an easy, you know, if you would say like, oh, hey, there’s this app on your phone.” (Parent 6) “If she had been exposed to it here… with other kids and therapists that she knew personally… I think she would buy into it.” (Parent 10)
Establishing evidence of effectiveness	“Oh yeah, she loves it. She loves engaging with it, especially when it gets more difficult, she likes being able to challenge herself to be able to pass more difficult songs on a more difficult level” (Parent 2) “If this was research funded and there are people that applied for it… that would be great.” (Parent 8)
Showcasing positive patient experiences	"Like educational, things that she learned when she went to pain clinic that she could learn or hear or have reinforced at home would be, would be terrific”. (Parent 1) “If you show them in appointments so parents can see it’s not mindless gaming, that’d help them believe it’s useful.” (Parent 2)
Theme: Program content and features	
Relaxation and mindfulness	“Like just to manage her pain, I don’t know, learn breathing techniques and relaxation techniques.” (Parent 3) “I guess, take your mind off of it… But if it was…just somebody either to visually walk you through something cool and amazing to like take your mind off. Kind of like what they do with like meditation.” (Parent 9)
Physical activity	“My husband had gotten a virtual reality headset and so we have one, and once he got that we started using that as part of her daily exercise.” (Parent 2) "There’s enough going on to help take the mind away from it. Yeah. And it might even make the person feel like they just maybe they got a workout in, right? Like there could be some positive. There’s going to be some endorphins and some things flowing around as a result of it. Maybe it’s enough to, you know, temporarily relieve pain.” (Parent 8)
Customization	"I think kind of like, well, even like roller coasters, I think would be fun because like that, that could be like something that they’re maybe have like a fear of or, you know, and that could help them get over the fear too and feel like they’re in control.” (Parent 7) “A way for the patients, like on their profiles to be able to create how their world is… Build their person like their avatar... To build their own theme park… These are my coping skills.” (Parent 11)
Social connection	“This would be cool. Yeah. If they could connect with other patients... like a virtual support group.” (Parent 11) “I think somebody like her age group going through the same thing. I think that would help.” (Parent 12)

a VR: virtual reality.

### Views Toward VR Technology for Chronic Pain Management

Overall, parents (n=11) expressed positive views toward integrating VR technology into pediatric and adolescent chronic pain management. Perceptions of using VR technology for chronic pain management were categorized into two subthemes: (1) perceived benefits and (2) distraction and redirection. The overwhelming majority of parents (n=11) reported that VR technology is a promising tool for at-home pain management, with over half of parents (n=7) emphasizing that VR technology may reduce pain and pain-related stress, improve functioning, and increase self-efficacy. Three parents also described how VR technology could limit missed school days and activities due to medical appointments. One parent said, *“*I think being able to do anything at home rather than take them to the doctor, or get in the car and drive or have to miss school, it’s like if it’s a treatment that’s even like 2% effective and you can do it at home easily without having to take time off work and travel I think that would be great” (Participant 1). Eight parents also reported that VR technology could be used as a method of distraction—shifting the child’s attention away from pain sensation and discomfort via engaging activities in an immersive, virtual environment.

### Barriers to Using VR Technology for Chronic Pain Management

Although parents reported positive views toward VR technology for pediatric and adolescent chronic pain management overall, nearly all parents (n=10) reported barriers to the use of VR technology. Barriers to the use of VR technology were categorized into four subthemes: (1) accessibility, (2) complexity of VR technology, (3) potential overuse of VR technology, and (4) discomfort and symptom exacerbation. Parents described several potential accessibility barriers—including financial barriers and lack of a personally owned VR headset. One parent said, “The only barrier that I could see is like, if insurance doesn’t cover that, finances, finances, like, yeah, because not everybody has a tablet or a phone or if it’s a full headset gear or whatever. Like not everybody has the ability to access that*”* (Participant 6). Another parent noted that not all families have access to the internet, so VR technology would need to be accessible offline. Some parents also reported that VR equipment and technology can be complicated and difficult to use. One parent stated, “…if you’re not technology savvy, it can be a little confusing to figure it out” (Participant 1). Others (n=3) noted that technical issues could arise that may discourage consistent use of VR. It was also recommended that developers “…make sure that it’s something that’s easy to use that a child could set up without the help of their parent” (Participant 1).

Several parents (n=4) expressed concerns that VR technologies would promote excessive screen time in children and adolescents with chronic pain, which may lead to decreased social capital and social isolation. One parent suggested the use of screen time limits to address this parental concern. A few parents (n=2) also reported that the use of VR technology may exacerbate symptoms and cause discomfort for some children depending on the type of pain they experience and what the VR technology entails, suggesting that not all children and adolescents with chronic pain will benefit from the use of VR technologies. For example, VR technology may worsen symptoms in children who experience chronic migraine due to factors like flashing lights and rapid movements. Participant 4 stated, “If it causes more pain with movement or certain moves they have to do to get through the game. I think it would probably just depend on where the pain is and what type of game it is.” Collectively, these barriers underscore the importance of designing VR technologies that are affordable, user-friendly, and adaptable to diverse pediatric pain conditions.

### Facilitators to Integrate VR Technology Into Chronic Pain Management

Participants had several recommendations for strategies to facilitate the integration of VR technology into pediatric and adolescent chronic pain management. Facilitators to the use of VR technology for chronic pain management were categorized into four subthemes: (1) addressing financial barriers, (2) integrating VR technology within clinical care, (3) demonstrating effectiveness, and (4) showcasing positive experiences of children and adolescents with chronic pain. Parents provided various recommendations to overcome financial barriers to the use of VR technology among children and adolescents with chronic pain. One parent suggested that health care professionals advocate for insurance companies to cover the cost associated with VR technologies for chronic pain management. Others suggested providing VR headsets to families at no cost or allowing families to rent headsets.

Further, parents noted that health care professionals should integrate VR technologies, if effective, into routine clinical care for children and adolescents with chronic pain. They suggested that introducing VR technologies during a clinical visit would enhance buy-in and compliance with the intervention and provide an opportunity for families to understand the benefits of VR technology and be trained in how to appropriately use the technology. A participant said, “I think incorporating it in appointments and visits so parents can see what the kid is doing and enjoy it. And you know seeing that it can be helpful and beneficial in a lot of ways instead of it just being like, you know just a mindless video game would be helpful” (Participant 2). Some parents (n=5) also emphasized the importance of establishing evidence of the effectiveness of VR technologies for pediatric and adolescent chronic pain management—including the impact of the intervention on pain, functioning, and mental health and well-being. Parents highlighted the value of showcasing positive experiences of the integration of VR technology into chronic pain management among children and adolescents. Five parents described how hearing positive experiences of VR technology use could enhance credibility and trust, provide valuable insights into real-world experiences, and promote patient uptake and engagement with VR technology.

### Recommendations for Program Content and Features

Parents provided recommendations for the content and features of future VR technologies for pediatric and adolescent chronic pain management. Recommendations for program content and features were categorized into 4 subthemes: physical activity and movement, relaxation and mindfulness techniques, social connection, and customizable content. Parents described how VR technologies have great potential to elevate physical, mental, and social health and well-being in children and adolescents with chronic pain. Six parents described how VR technology could improve levels of physical function and physical activity. Parents discussed how VR technology could incorporate adaptable exercises or therapeutic movements, enabling children and adolescents with chronic pain to stay physically active. Participant 10 said, “anything that sort of gamified the exercises that she needed to be doing helped her a lot.” Another parent suggested that future VR technologies incorporate physical therapy activities used in rehabilitation or enable children and adolescents to connect with their physical therapist in the virtual environment to promote adherence with treatment plans.

Moreover, many parents (n=8) discussed how VR technology could be used to help children and adolescents practice relaxation, meditation, or mindfulness—recognizing that these evidence-based mental health practices can help children and adolescents cope with their pain and pain-related anxieties. Some parents (n=6) highlighted how VR technology that can deliver mindfulness practices through a guided audio voiceover may enhance therapeutic effectiveness and the use of mindfulness practice. One parent said, “Having somebody sit there and say, I need you to close your eyes, and I need you to do this…or listen or let me take you on a visual journey on the, on the VR or something… I personally think that they would use it all the time” (Participant 10). Other parents (n=6) suggested that future VR technology for chronic pain management incorporate guided breathing exercises or calming visuals to help children and adolescents with chronic pain relax, potentially alleviating the sensation and distress of pain. Parents also described how future VR technologies could provide a safe and welcoming environment for children and adolescents with chronic pain to connect with others experiencing chronic pain. Parents shared that the ability to connect with other children with similar experiences and challenges may help reduce feelings of isolation and promote empathy and understanding. In total, 2 parents also mentioned that the VR environment should be personalized to meet the needs of the child with chronic pain.

## Discussion

### Principal Findings

This study provided data to better our understanding of whether and how parents envision using VR technology to enhance at-home pain management for children or adolescents with chronic pain. The findings revealed favorable attitudes toward the use of VR technology in pediatric and adolescent chronic pain management, with many parents expressing excitement toward the emergence of new digital health technologies to optimize pain care. Our findings illuminate the perceived benefits of VR technologies for children or adolescents with chronic pain—including the potential to incorporate relaxation and mindfulness exercises, support physical activity, and foster social connections among children and adolescents with similar health experiences. These findings extend past studies demonstrating the effectiveness of VR-based mindfulness interventions in reducing symptoms of anxiety or depression and alleviating pain in children and young adults with chronic pain [28] or other chronic conditions such as inflammatory bowel disease [29], cancer [28], or polycystic ovary syndrome [28]. More research is required to support the effectiveness of VR technologies for chronic pain management in children and adolescents.

Importantly, the study identified key facilitators and barriers to the use of VR technology for at-home chronic pain management in children or adolescents. Integrating VR technologies within clinical care emerged as a major facilitator to the use of such technology among children or adolescents with chronic pain. This finding is critical to health care professionals as it highlights the importance of health care providers discussing potential digital health technologies for chronic pain management with children and their families. Engaging patients and their families in discussions about the use of digital health technologies for pain management may empower patients to take ownership of their chronic pain management plan and, in turn, maximize patient engagement and adherence. Establishing evidence of effectiveness is also a key factor when considering implementing VR technology into chronic pain management. Despite growing research on the benefits of integrating digital health technologies into chronic pain management [30], robust data supporting the use of VR technology for children or adolescents with chronic pain are lacking. Future studies that examine children and adolescents’ acceptance of VR technology in pediatric and adolescent chronic pain treatment plans based on the TAM framework would better our understanding of the potential acceptance or rejection of such technologies. Parents also indicated that showcasing positive patient experiences could, potentially, increase children’s buy-in and engagement with VR technologies for chronic pain management.

Although parents expressed overwhelming support for the use of VR technology for chronic pain management in children or adolescents, major barriers—including economic, physical, and technical challenges—were also identified. Parents indicated that addressing economic barriers to access, such as the high cost of VR headsets, is key to VR adoption. They suggested that health care professionals and researchers advocate for insurance companies to cover the costs of VR headsets. To address these challenges, it is essential that state regulatory and payer policies are aligned with evidence-informed care for chronic pain management, placing fewer limitations on when and what types of digital health interventions are covered and at what rates [31]. Health care systems could also consider implementing triage processes to identify children or adolescents who need to be prioritized based on the desired outcome (eg, expected success of VR) or on patient characteristics (eg, pain condition) and ensuring they have optimal access to VR technology. Pediatric and adolescent pain rehabilitation programs could consider implementing loaner programs for children or adolescents to borrow VR headsets during their treatment or partnering with local organizations such as schools or libraries that offer a VR headset loan program. Additionally, VR developers should design VR interventions for chronic pain management that can work on low-cost devices or on mobile VR.

Another key barrier identified was the potential for discomfort or symptom exacerbation, especially in children or adolescents with chronic migraines and dizziness. Given these concerns and the risk of motion sickness in VR, the use of VR may not be appropriate for all children and adolescents with chronic pain. Future studies should explore whether parental views toward the application of VR technology to chronic pain management differ by pain complaint. Youth may also experience difficulties navigating complex virtual environments in VR, which may limit the adoption or effectiveness of VR technologies in chronic pain relief. It is important that VR technology for chronic pain management is easy to use and that children and adolescents with chronic pain and their families receive training on how to use the technology prior to integrating it within the child’s pain treatment plan. Some parents also expressed concerns with excessive VR use, which may lead to disconnection from real-world activities.

These findings are consistent with prior studies documenting the barriers of VR use [32,33]. For example, Elser et al [32] found that environmental factors, such as the high cost of VR devices and technical limitations in device performance, are commonly reported barriers to VR interventions for chronic pain. Moreover, Sarkar et al [33] identified reimbursement challenges and difficulties with workflow integration as significant barriers to implementing VR in clinical settings. Addressing these issues is critical to ensuring that VR technologies can be effectively and equitably deployed for pain management. It is clear from parents’ comments that these barriers need to be addressed to minimize access disparities and maximize the effectiveness of VR technology.

### Implications and Future Research

Our findings provide important insight into parents’ perceptions of barriers, facilitators, and recommendations related to integrating VR technologies into pediatric and adolescent chronic pain management. The findings of our study have important implications for developing VR technologies for pediatric and adolescent chronic pain management and future research studies and provide insight into the potential acceptance or rejection of such technologies. First, VR technologies may be a nonpharmacological means of managing anxiety, depression, and other mental health disorder–related symptoms in children and adolescents with chronic pain. As such, future VR technologies for children and adolescents with chronic pain should consider including mindfulness and meditation activities. Incorporating such activities into future VR technologies has the potential to empower children and adolescents to manage negative or worrisome thoughts about their pain and promote acceptance of physical sensations, thus, in turn, reducing stress and alleviating pain. Health care professionals could also harness VR technologies to provide extended support or booster sessions to reinforce techniques or skills learned in multidisciplinary pain management programs, such as relaxation techniques, adaptive coping mechanisms, or cognitive behavioral therapy strategies. Extended support or booster sessions may enhance the application of taught techniques or skills, which may alleviate pain and anxiety, depression, or other mental health disorder–related symptoms.

There is a unique opportunity for VR technologies to enable children and adolescents with chronic pain to escape their restricted physical realities and engage in physical activities in a safe, immersive, and engaging environment. Parents in our study suggested that the interactive nature of VR technologies may temporarily distract children or adolescents from the pain and potentially negative thoughts they are experiencing, enabling them to engage in informal exercise in a fun, relaxed, and safe way. Studies show that VR technologies can make physical activity more enjoyable for children and adolescents, leading to increased motivation and adherence to physical therapy regimes [34-36]. As such, future VR technologies should consider embedding some of the following features to promote physical activity in children or adolescents with chronic pain: (1) gamification, turning physical activity into game-like experiences with challenges, rewards, and progression systems to increase engagement and motivation for exercise; (2) personalized avatars and real-time feedback on one’s movements, which may foster a sense of accomplishment and ensure proper form; and (3) adaptability for different fitness levels, enabling youth of all abilities to participate. These types of VR features have been used effectively to promote physical activity and rehabilitation in other populations of children and adolescents, such as burn survivors [37] and those with cerebral palsy [38-40] or a developmental disability [41,42].

Rigorous research is needed to guide the evolution of VR technologies for pediatric and adolescent chronic pain management and to ensure that children and adolescents and their families perceive the technology to be useful and easy to use. Future research should consider the use of co-design approaches to involve key stakeholders—including children and adolescents with chronic pain and their families and health care providers—in the design of the VR technology. For example, researchers, in collaboration with clinicians and VR designers, could facilitate interactive workshops with children and adolescents with chronic pain and their caregivers to understand the problem, define the need for VR technologies for chronic pain management, and develop the initial design and content of such novel technologies. Prior studies show that the use of co-design approaches helps create more user-centered and stakeholder-supported technologies [43-45]. New VR technologies should undergo usability testing early in the development process to identify potential issues and pain points, ultimately leading to user-friendly technology that meets the unique needs of children and adolescents with chronic pain and provides positive experiences for users. Once VR technologies are created, studies will be required to determine the effectiveness of new VR technologies in reducing pain and improving psychosocial health outcomes in children and adolescents.

### Limitations

It is important to note the limitations of this study. First, the sample was lacking in diversity, consisting almost exclusively of mothers of daughters with chronic pain. However, this demographic composition is typical of the chronic pain population given the higher prevalence of chronic pain in girls as compared to boys [1]. Mothers are also more commonly the primary caregivers of children and adolescents with chronic pain as compared to fathers [46]. Second, all participants were recruited from pain management services at a large Midwest children’s hospital, and it is possible that the views of parents of children with chronic pain with less access to specialized pain management services or specific pain conditions may differ. Our findings likely do not represent the full range of parents’ views toward the integration of VR technology into chronic pain management. Future qualitative studies garnering the perspectives of a larger and more diverse sample of parents of children with chronic pain are needed to better our understanding of the perceived acceptability and potential applications of VR technology for chronic pain management in children and adolescents.

Third, participants were recruited through purposive sampling, which may introduce researcher bias. Further, our results may be subject to social desirability bias, as the parents may have provided positive views toward VR technology as they thought this was what the interviewer wanted to hear. To mitigate social desirability bias, the interviewer implemented various strategies to encourage more honest and authentic responses—including using neutral language, ensuring privacy and confidentiality, and emphasizing that there are no right or wrong answers. Another limitation is that we explored parents’ views toward the integration of VR into chronic pain management as opposed to the views of children and adolescents with chronic pain themselves. Parents may lack accurate insight into their child’s experiences and views toward VR technology for pain management, and the child’s voice may be lost or underrepresented. Future studies are critically needed to explore children and adolescents’ perspectives on the use of VR in chronic pain management plans and to co-design such technologies for chronic pain management in adolescents using human-centered design methodologies. Finally, participants’ understanding of VR was based solely on their prior knowledge of and experiences with VR technology. The results of this study should be interpreted with these limitations in mind. Yet, despite these limitations, this study provides important and unique insight into parents’ perspectives regarding the potential use of VR technology in pediatric and adolescent chronic pain management plans and emphasizes the necessity of larger studies that further investigate multiple stakeholders’ perspectives.

### Conclusions

This study suggests that VR technology offers great potential for chronic pain management in children and adolescents, and such technology would be well received and adopted by children and their families. Developing VR digital interventions incorporating mental health promotion strategies, physical activities, and social connections may enhance the ability of children with chronic pain to perform activities of daily living, maintain social relationships, and enjoy a good quality of life. However, there are several barriers that need to be addressed to enhance the accessibility of VR technology for use in pediatric and adolescent chronic pain management and promote equity in care. We provided practical suggestions to guide the development and adoption of effective and equitable VR technology for chronic pain management in children and adolescents and to inform future studies.

## Abbreviations

RATS: Relevance, Appropriateness, Transparency, Soundness
TAM: technology acceptance model
VR: virtual reality

## References

[1] Chambers CT, Dol J, Tutelman PR (2024). The prevalence of chronic pain in children and adolescents: a systematic review update and meta-analysis. Pain.

[2] Pico M, Matey-Rodríguez C, Domínguez-García A, Menéndez H, Lista S, Santos-Lozano A (2023). Healthcare professionals’ knowledge about pediatric chronic pain: a systematic review. Children (Basel).

[3] Manworren RCH (2019). Pediatric chronic pain. Pediatric pain PRN curriculum. https://www.luriechildrens.org/globalassets/media/pages/for-healthcare-professionals/prn-curriculum/core/chronic/april2020/2020.pediatricprn.chronicpain.final.pg.pdf.

[4] Landry BW, Fischer PR, Driscoll SW (2015). Managing chronic pain in children and adolescents: a clinical review. PM R.

[5] Emerson ND, Bursch B (2020). Communicating with youth about pain: developmental considerations. Children (Basel).

[6] Eccleston C, Malleson PN, Clinch J, Connell H, Sourbut C (2003). Chronic pain in adolescents: evaluation of a programme of interdisciplinary cognitive behaviour therapy. Arch Dis Child.

[7] Peng P, Stinson JN, Choiniere M (2007). Dedicated multidisciplinary pain management centres for children in Canada: the current status. Can J Anaesth.

[8] Liossi C, Howard RF (2016). Pediatric chronic pain: biopsychosocial assessment and formulation. Pediatrics.

[9] Domhardt M, Schröder A, Geirhos A, Steubl L, Baumeister H (2021). Efficacy of digital health interventions in youth with chronic medical conditions: A meta-analysis. Internet Interv.

[10] Griffin A, Wilson L, Feinstein AB (2020). Virtual reality in pain rehabilitation for youth with chronic pain: pilot feasibility study. JMIR Rehabil Assist Technol.

[11] Jibb LA, Stinson JN, Stevens BJ Oxford Textbook of Pediatric Pain.

[12] Tas FQ, van Eijk CAM, Staals LM, Legerstee JS, Dierckx B (2022). Virtual reality in pediatrics, effects on pain and anxiety: a systematic review and meta-analysis update. Paediatr Anaesth.

[13] Won AS, Bailey J, Bailenson J, Tataru C, Yoon IA, Golianu B (2017). Immersive virtual reality for pediatric pain. Children (Basel).

[14] Goudman L, Jansen J, Billot M (2022). Virtual reality applications in chronic pain management: systematic review and meta-analysis. JMIR Serious Games.

[15] Hess CW, Rosenbloom BN, Mesaroli G (2025). Extended reality (XR) in pediatric acute and chronic pain: systematic review and evidence gap map. JMIR Pediatr Parent.

[16] Simons LE, Hess CW, Choate ES (2022). Virtual reality-augmented physiotherapy for chronic pain in youth: protocol for a randomized controlled trial enhanced with a single-case experimental design. JMIR Res Protoc.

[17] Hemphill S, Rodriguez S, Wang E (2022). Virtual reality augments movement during physical therapy: a pragmatic randomized trial. Am J Phys Med Rehabil.

[18] Bexson C, Oldham G, Wray J (2024). Safety of virtual reality use in children: a systematic review. Eur J Pediatr.

[19] Davis FD, Al-Suqri MN, Al-Aufi AS (2015). Information Seeking Behavior and Technology Adoption: Theories and Trends.

[20] Holden RJ, Karsh BT (2010). The technology acceptance model: its past and its future in health care. J Biomed Inform.

[21] Yarbrough AK, Smith TB (2007). Technology acceptance among physicians: a new take on TAM. Med Care Res Rev.

[22] Curry LA, Nembhard IM, Bradley EH (2009). Qualitative and mixed methods provide unique contributions to outcomes research. Circulation.

[23] Creswell JW, Poth CN (2025). Qualitative Inquiry & Research Design: Choosing Among Five Approaches.

[24] Clark JP, Godlee F, Jefferson T (2003). Peer Review in Health Sciences.

[25] Graneheim UH, Lindgren BM, Lundman B (2017). Methodological challenges in qualitative content analysis: a discussion paper. Nurse Educ Today.

[26] Guest G, Bunce A, Johnson L (2006). How many interviews are enough?. Field Methods.

[27] Hennink M, Kaiser BN (2022). Sample sizes for saturation in qualitative research: a systematic review of empirical tests. Soc Sci Med.

[28] Hughes O, Shelton KH, Penny H, Thompson AR (2023). Living with physical health conditions: a systematic review of mindfulness-based interventions for children, adolescents, and their parents. J Pediatr Psychol.

[29] Wren AA, Neiman N, Caruso TJ (2021). Mindfulness-Based Virtual Reality Intervention for Children and Young Adults with Inflammatory Bowel Disease: A Pilot Feasibility and Acceptability Study. Children (Basel).

[30] Keogh E, Rosser BA, Eccleston C (2010). e-Health and chronic pain management: current status and developments. Pain.

[31] Burstin H, Clark KJ, Duff N (2023). Integrating telehealth and traditional care in chronic pain management and substance use disorder treatment: an action agenda for building the future state of hybrid care. NAM Perspect.

[32] Elser A, Lange M, Kopkow C, Schäfer AG (2024). Barriers and facilitators to the implementation of virtual reality interventions for people with chronic pain: scoping review. JMIR XR Spatial Comput.

[33] Sarkar U, Lee JE, Nguyen KH, Lisker S, Lyles CR (2021). Barriers and facilitators to the implementation of virtual reality as a pain management modality in academic, community, and safety-net settings: qualitative analysis. J Med Internet Res.

[34] Farič N, Yorke E, Varnes L (2019). Younger adolescents’ perceptions of physical activity, exergaming, and virtual reality: qualitative intervention development study. JMIR Serious Games.

[35] Mouatt B, Smith AE, Mellow ML, Parfitt G, Smith RT, Stanton TR (2020). The use of virtual reality to influence motivation, affect, enjoyment, and engagement during exercise: a scoping review. Front Virtual Real.

[36] Phelan I, Carrion-Plaza A, Furness PJ, Dimitri P (2023). Home-based immersive virtual reality physical rehabilitation in paediatric patients for upper limb motor impairment: a feasibility study. Virtual Real.

[37] Basha MA, Aboelnour NH, Aly SM, Kamel FAH (2022). Impact of kinect-based virtual reality training on physical fitness and quality of life in severely burned children: a monocentric randomized controlled trial. Ann Phys Rehabil Med.

[38] Jha KK, Karunanithi GB, Sahana A, Karthikbabu S (2021). Randomised trial of virtual reality gaming and physiotherapy on balance, gross motor performance and daily functions among children with bilateral spastic cerebral palsy. Somatosens Mot Res.

[39] Arnoni JLB, Kleiner AFR, Lima CRG, de Campos AC, Rocha N (2021). Nonimmersive virtual reality as complementary rehabilitation on functional mobility and gait in cerebral palsy: a randomized controlled clinical trial. Games Health J.

[40] Malick WH, Butt R, Awan WA, Ashfaq M, Mahmood Q (2022). Effects of augmented reality intervention on the range of motion and muscle strength of upper extremity in children with spastic hemiplegic cerebral palsy: a randomized clinical trial. Games Health J.

[41] Lee HK, Jin J (2024). Combined virtual-reality- and gym-based physical activity intervention for children with a developmental disability: effects on physical activity levels, motor skills, and social skills. Adapt Phys Activ Q.

[42] Ju YJ, Du YC, Huang HC, Hu Kao PS, Cherng RJ (2023). Development and feasibility of a virtual reality-based exergaming program to enhance cardiopulmonary fitness in children with developmental coordination disorder. Front Pediatr.

[43] Bombard Y, Baker GR, Orlando E (2018). Engaging patients to improve quality of care: a systematic review. Implement Sci.

[44] Durall E, Bauters M, Hietala I, Leinonen T, Kapros E (2019). Co-creation and co-design in technology-enhanced learning: Innovating science learning outside the classroom. IxD&A.

[45] Grindell C, Coates E, Croot L, O’Cathain A (2022). The use of co-production, co-design and co-creation to mobilise knowledge in the management of health conditions: a systematic review. BMC Health Serv Res.

[46] Jordan A, Crabtree A, Eccleston C (2016). “You have to be a jack of all trades”: fathers parenting their adolescent with chronic pain. J Health Psychol.

